# Sex differences in global metrics of brain size across the lifespan

**DOI:** 10.3389/fnins.2026.1646144

**Published:** 2026-02-27

**Authors:** Samuel N. Vucic, Brianna Georges, Sophia Frangou, Neda Sadeghi, Tonya White

**Affiliations:** 1 Section on Social and Cognitive Developmental Neuroscience, National Institute of Mental Health, National Institutes of Health, Bethesda, MD, United States; 2 Department of Psychiatry, University of British Columbia, Vancouver, BC, Canada; 3 Department of Psychiatry, Icahn School of Medicine at Mount Sinai, New York, NY, United States

**Keywords:** brain morphometry, Cohen’s d, head circumference, lifespan, sex differences, structural MRI

## Abstract

**Introduction:**

While global brain volume differences between males and females have been shown to manifest during prenatal life, it is unclear whether global differences remain stable or show variability over the lifespan. Therefore, our goal was to use the existing literature coupled with large-population-based studies to assess age-related differences in effect size estimates of brain size between males and females over the life-span.

**Methods:**

We quantified effect size measures (Cohen’s d) of sex differences in terms of head circumference using data drawn from the literature of prenatal (14 weeks to birth) ultrasounds of *n* = 36,487 uncomplicated healthy births and direct postnatal (0–7 years) head circumference measurements from 85,598 children. The effect size of sex differences of cortical surface area, cortical thickness, and cortical volume were also computed from structural magnetic resonance imaging data from 25,846 healthy individuals aged 5–89 years.

**Results:**

Head circumference was consistently larger in males from fetal life through early childhood, with effect sizes typically ranging from ∼0.3 to 0.5 across studies and developmental stages. Males exhibited greater surface area and cortical volume across development, with effect sizes increasing from ∼0.4 at age 5 to ∼1.4 by age 24, after which they remained relatively stable. Cortical thickness showed a female advantage during childhood that diminished by mid-adolescence.

**Conclusion:**

The effect size of sex differences in global brain metrics does not remain constant across the lifespan. The underlying mechanisms are likely to involve endocrine and other neurodevelopmental processes. Future studies, especially preclinical and longitudinal studies beginning in the prenatal period may offer insight into the underlying mechanisms and the potential for translation of these findings, assessing the curves in patients with neurodevelopmental disorders.

## 1 Introduction

Prior literature has demonstrated sex-based differences in global measures of brain size using different measurement techniques (Bethlehem et al., 2022; Gennatas et al., 2017; Ritchie et al., 2018; Ruigrok et al., 2014). Head circumference (HC) is an easy-to-measure external proxy for overall brain size in pediatric populations, as studies have shown high correlations between HC with both CT and MRI measures (Bartholomeusz et al., 2002; Hshieh et al., 2016; Lindley et al., 1999). Larger HC reflects a larger cranial vault, which closely approximates intracranial volume (ICV), which is the total space occupied by brain tissue, cerebrospinal fluid, and vasculature. Cortical volume constitutes a substantial portion of ICV; it scales with ICV but the correspondence is imperfect as cortical volume is influenced by multiple factors (e.g., pruning, neurodegeneration) that do not affect ICV or HC. In this study we focus on HC during early development (mid-gestation and early childhood period) and on cortical volume in healthy individuals aged 5–89 years (childhood to late adulthood). The rationale for this two tier strategy is justified by the availability of data during these periods of life. Specifically, HC is routinely collected proxy for global brain size in fetal life and early childhood. HC correlates strongly (*r* > 0.9) with ICV in older adults (Hshieh et al., 2016), and head circumference has been demonstrated to predict total brain volume well within neonates (Lindley et al., 1999) and in healthy children 1.7–6 years old (Bartholomeusz et al., 2002). HC can be obtained serially at prenatal ultrasound and well-baby visits, and is typically embedded in pediatric growth surveillance. Capturing HC from mid-gestation to age 7 years therefore furnishes dense, population-representative data of early neurodevelopment with minimal risk and expense. While challenging in very young children, the use of MRI can yield high-resolution estimates of brain morphometry, including precise measures of global cortical volume, surface area, and thickness.

Sex differences have been reported in most measures of brain size and cortical morphometry (Bethlehem et al., 2022; Gennatas et al., 2017; Ritchie et al., 2018; Ruigrok et al., 2014). A great deal of this literature compares neuroimaging metrics or head circumference between sexes (Bethlehem et al., 2022; Gennatas et al., 2017; Ritchie et al., 2018), with large public health organizations including the Centers for Disease Control (World Health Organization [WHO], 2007) and World Health Organization (Kuczmarski et al., 2002) collecting morphometric measures (e.g., head circumference growth curves) representative of the pediatric population. Researchers have also examined sex differences in prenatal ultrasound data (Galjaard et al., 2019; Melamed et al., 2013).

While such sex differences in brain morphology are present, consideration must be given to whether these differences relate to overall growth trajectories involving brain and body size, whether the differences are brain specific, or a combination of the two. Gross morphological sex differences in the brain may underlie the genetic and hormonal influences related to sex-specific physiological development in humans. Eliot et al. (2021) provided analysis suggesting that only 1% of observed regional male-female brain differences survived correction for body size. This paper additionally suggests that “larger bodies require larger brains and the [sex/gender] difference in brain volume mostly parallels the divergence of male/female body size during development.” Eliot et al. (2021) cites a longitudinal study of 387 adolescents, finding that “the [sex/gender] difference in TBV grew steadily from 6% difference at age 7 to a 15% difference at age 20, in parallel with the divergence in height and weight over adolescence” (Lenroot et al., 2007; Paus et al., 2017).

Studies have shown that global brain size and sex covariates variably explain compartmental or regional sex differences in metrics of brain volume (Jäncke et al., 2015; Lüders et al., 2002), although regional brain differences are outside of the scope of this paper. In any case, we believe that even global differences which can be explained by normal developmental height and weight might be considered meaningful to both a fundamental understanding of the brain and sex differences, and potential applications of our analyses.

Some literature quantifies effect size with one value across a broad age range. Ruigrok et al. (2014), for example, found global effect sizes ranging from 1.68 for the cerebellum to 3.35 for the cerebrum. Relatedly, a great deal of work also exists that maps MRI measures in population-level age-related differences across the lifespan, even distinguishing trajectories for sex (Bethlehem et al., 2022; Tomoto et al., 2023). For example, Bethlehem et al. (2022) presents trajectories of a number of global brain metrics (total cerebrum volume, total surface area, etc.) as “*raw, non-centiled data*; population trajectories of the median” in males and females. Our paper, however, presents sex-based effect size population trajectories using mean head circumference, surface area, and cortical volume. So, while the Bethlehem et al. (2022) paper presents population trajectories of global brain metrics in both males and females, our paper assesses differences between males and females using effect size-based trajectories. Surprisingly, however, there has been a relative dearth of investigation into developmental trajectories of sex-based effect sizes in global brain metrics in healthy or clinical populations.

Further, many psychiatric and neurodevelopmental conditions differ between males and females with respect to prevalence, average age of onset, and symptom profiles (Bao and Swaab, 2010; Bölte et al., 2023; Paus et al., 2008). Conditions with greater female prevalence include depressive disorders, anxiety disorders, post-traumatic stress disorder, and eating disorders; while conditions with greater male prevalence include autism spectrum disorder (ASD), several substance use disorders, attention deficit/hyperactivity disorder (ADHD), dyslexia, and conduct disorder (Bao and Swaab, 2010; Eaton et al., 2012). Major depression, a number of anxiety disorders and post-traumatic stress disorder (Serra-Blasco et al., 2021), anorexia nervosa (Bracké et al., 2023), ADHD (Hoogman et al., 2019; Mous et al., 2014; Shaw et al., 2018), and ASD (Blanken et al., 2015; Blanken et al., 2018) have also been suggested to have patient/control differences in global or regional brain morphology. Examining sex difference trajectories in global brain morphometry, along with potential underlying influences on such differences may serve to illuminate mechanistic and clinical understanding of sex differences in psychiatric and neurodevelopmental conditions.

An additional benefit of a lifespan approach of assessing effect sizes between males and females is related to better understand temporal variations that contribute to prediction algorithms. Research into machine learning and deep learning has revealed high model accuracy in predicting sex using T_1_-weighted structural MRI of the brain in typically-developing children and adolescents (Bi et al., 2023; Mendes et al., 2021; Sepehrband et al., 2018). Convolutional neural network (CNN) based sex prediction using longitudinal structural brain imaging measures has been shown to vary in accuracy between participants from the ABCD dataset at baseline (9–10 years), and 2-year follow up (Bi et al., 2023), with a higher mean accuracy at the 2-year follow-up period (roughly 97.5% compared to 97.2% at baseline—a significant improvement at *p* = 0.0325). Factors that might contribute to age-related differences in accuracy are unclear. Therefore, identifying age-related differences in sex-based effect size measures of global brain measures can inform studies of sex classification at any age across the lifespan.

In this study, we quantified the effect sizes of sex differences in HC, cortical surface area, cortical thickness, and cortical volume in multiple datasets spanning from prenatal life through old age. Sex differences were quantified through Cohen’s d effect sizes, using females as the reference. We used ultrasound-based head circumference data in the prenatal period (14 weeks gestational age to birth), head circumference measures in the postnatal period (0–7 years), and MRI-based measures of cortical surface area, cortical thickness, and cortical volume from 5 to 89 years of age.

## 2 Materials and methods

Our datasets comprised a total sample of 139,999 healthy subjects from 41 independent datasets. Datasets spanned three lifespan periods; including: (i) fetal (prenatal) period; (ii) early childhood; (iii) and early-childhood to late adulthood (MRI data). Of the total sample size, 28,555 were human fetuses between 13 and 40 weeks gestational age with data extracted from summary data from 4 studies (Galjaard et al., 2019; Melamed et al., 2013; Schwärzler et al., 2004; Yeo et al., 2017), 85,598 children between 0 and 5 years-of-age with data extracted from summary data from 3 studies (Bi et al., 2023; Jäncke et al., 2015; Paus et al., 2017), and 25,846 were participants between 5 and 89 years-of-age with individual MRI scans from studies participating in the 33 studies listed in Table 1.

**TABLE 1:** Late childhood to late adulthood.

Imaging database	n	Age range (years)	M/F ratio	Access request
ABCD	3,759	8–11	0.90	ABCD
ABIDE I	438	6–21	4.28	ABIDE I
ABIDE II	433	5–21	2.07	ABIDE II
ADHD-200	389	7–21	0.87	ADHD-200
Ann Arbor a	24	13–41	7.00	FCON
Ann Arbor b	33	19–80	0.83	FCON
Atlanta	28	22–57	0.87	FCON
Baltimore	23	20–40	0.53	FCON
Bangor	20	19–38	Females only	FCON
Beijing Zang	198	18–26	0.62	FCON
Berlin Marguiles	26	23–44	1.00	FCON
Cam-CAN	643	18–89	0.95	CAMCAN
Cambridge	198	18–30	0.61	FCON
HCP aging	1,838	36–89	0.82	HCP aging
HCP development	652	5–22	0.86	HCP development
Healthy brain network/CMI	214	5–21	1.23	HBN/CMI
ICBM	85	19–85	0.89	FCON
Imagen	1,840	13–16	0.96	IMAGEN
Leiden_2180	12	20–27	Females only	FCON
Leiden_2200	19	18–28	1.38	FCON
Milwaukee_b	46	44–65	0.48	FCON
Munchen	16	63–74	1.67	FCON
Newark	19	21–39	0.67	FCON
New York_b	20	18–46	0.67	FCON
NYU_TRT	25	22–49	0.90	FCON
Orangeburg	20	25–55	3.00	FCON
Oulu	103	20–23	0.56	FCON
Oxford	22	20–35	1.20	FCON
Palo Alto	17	23–39	0.13	FCON
Queensland	19	23–34	1.38	FCON
Rockland	140	6–21	1.22	FCON
Saint Louis	31	21–29	0.50	FCON
UK Biobank	14,496	45–82	1.23	UK Biobank
Total	25,846	5–90	1.30 (avg)	

Generally, data sources were selected in accordance with data availability and large sample size. The studies of prenatal head growth were obtained through a literature search using terms that include; “prenatal,” “ultrasound,” “sex differences,” “growth,” “occipito-frontal circumference,” “OFC.” The reference lists of identified papers were also reviewed with the goal of obtaining all published papers on the topic. Head circumference datasets were obtained from three large-scale growth charts, those from the CDC, WHO, and the Chinese 4th National Survey on the Physical Growth and Development of Children (NSPGDC). MRI data was obtained from independent imaging samples that had been published by the ENIGMA-Lifespan working group for a systematic evaluation of brain-age prediction (Yu et al., 2024).

The sources we reference collected data either on the demographic variable of sex, or the use of gender at a time when these terms tended to be used interchangeably (National Academies of Sciences Engineering Medicine, 2022). While not explicitly stated, sex was determined by ultrasound classification in the prenatal studies. In the early childhood and MRI studies, data on sex was collected using demographic questionnaires administered to caregivers or participants at the time of their enrollment.

### 2.1 Head circumference

Head circumference (HC) from mid-gestation (11–12 weeks) to birth was measured using ultrasound in 4 independent studies that used routinely acquired data from uncomplicated live births (Table 2).

**TABLE 2:** Prenatal demographics.

Authors	n	Age range (gest. weeks)	n M/F	M/F ratio	Reference
Galjaard et al. (2019)	9,413	12–40	4,900/4,513	1.09	(Galjaard et al., 2019)
Melamed et al. (2013)	12,132	15–42	6,478/5,654	1.15	(Melamed et al., 2013)
Schwärzler et al. (2004)	5,055	15–40	2,589/2,466	1.05	(Schwärzler et al., 2004)
Yeo et al. (2017)	1,955	11–39	1,028/927	1.11	(Yeo et al., 2017)
Total	28,555	11–40	14,995/13,560	1.11	

Relevant literature demonstrates strong inter-observer and intra-observer agreement for the measure of cranial ultrasound, with both intraclass and interclass coefficients consistently estimated to be > 0.952 (Matthew et al., 2018) and measured in the second trimester to be as high as 0.996 and 0.995, respectively (Perni et al., 2004). While being distinct modalities, it is important to note that literature also demonstrates agreement between head circumference measurement by prenatal ultrasound, direct measurement, and MRI. For example, prenatal sonographic head circumference and direct measures of head circumference at birth show statistically significant agreement (R_*s*_ = 0.865, *p* < 0.001) (Gafner et al., 2020), and another study cites volumetric agreement in assessment of the fetal brain by ultrasound and MRI, outside the brain stem, intracranial volume, and growth plate (*p* < 0.001) (Wyburd et al., 2024).

Four studies included measures of HC in utero in males and females from large ultrasound databases (Galjaard et al., 2019; Melamed et al., 2013; Schwärzler et al., 2004; Yeo et al., 2017). Two studies were longitudinal and two had cross-sectional designs. Galjaard et al. selected between 1 and 4 timepoints per pregnancy while Schwarzler et al. selected three timepoints, one in each trimester. While longitudinal designs were present in these studies, in our analysis we calculated effect size measures within specific time epochs (per week). Each dataset had a nearly equal number of males and females (Table 2). Keen and Pearse (1988) was initially included from a literature search identifying prenatal head circumference-based studies, but excluded due to data being taken from spontaneous abortions rather than *in-utero* ultrasounds. The four studies assessed normal singleton pregnancies.

In the prenatal studies above we extracted the sex-specific mean/median and standard deviation/percentile measures of HC by age provided by the corresponding publication. The fetal ultrasound study by Melamed et al. (2013) alternatively provided sex-specific regression models for mean values and standard deviations of head circumference. Table 3 elaborates on the details of data type, data timepoint interval, and effect size calculation per data source.

**TABLE 3:** Additional data characteristics.

Data source	Data type	Timepoint interval	Extracted statistics for effect size calculation
Galjaard et al. (2019)	Sex-specific GAMLSS longitudinal regression of head circumference by age	Gestational week	Male and female ultrasound head circumference mean (estimated from 50th percentile) and standard deviation, by gestational week
Melamed et al. (2013)	Sex-specific regression model of head circumference by age	Gestational week	Male and female ultrasound head circumference mean and standard deviation (both estimated from relevant regression equations), by gestational week
Schwärzler et al. (2004)	Sex-specific head circumference centile charts (mean) by age	Gestational week	Male and female ultrasound head circumference mean (estimated from 50th percentile) and standard deviation, by gestational week
Yeo et al. (2017)	Sex-specific head circumference centile charts (mean) by age	Gestational week	Male and female ultrasound head circumference mean (estimated from 50th percentile) and standard deviation, by gestational week
WHO multicenter growth reference study (MGRS)	Sex-specific head circumference centile charts (median) by age	Month	Male and female head circumference mean (estimated from 50th percentile) and standard deviation, by month
CDC 2000 growth charts	Sex-specific head circumference centile charts (mean) by age	Month	Male and female head circumference mean (estimated from 50th percentile) and standard deviation, by month
The 4th national survey on physical growth and development of children (NSPGDC)	Sex-specific head circumference centile charts (mean) by age	Month	Male and female head circumference mean (estimated from 50th percentile) and standard deviation, by month
All MRI data	Raw, age-specific data of male and female ICV, cortical thickness and surface area	Cross sectional data of a variety of participants at various ages	Binning of data into windows with a size of 4 years and an incremental step of 2 years. Calculation of male and female mean and standard deviation within windows.

Data on HC from birth to the age of 7 years were obtained from three population-based growth-reference cohorts (Table 4): the WHO Multicentre Growth Reference Study (MGRS) (Paus et al., 2017), the U.S. CDC Growth Charts project (Jäncke et al., 2015), and the 4th National Survey on the Physical Growth and Development of Children (NSPGDC) (Bi et al., 2023).

**TABLE 4:** Postnatal demographics.

Reference study	N	Age range	n M/F	M/F ratio	Reference
WHO multicenter growth reference study (MGRS)	8,406	0–71 Months	4,344/4,062	1.07	(World Health Organization [WHO], 2007)
CDC 2000 growth charts	7,432	0–5 Years	3,803/3,629	1.05	(Kuczmarski et al., 2002)
The 4th national survey on physical growth and development of children (NSPGDC)	69,760	0–7 Years	34,901/34,859	1.00	(Zong and Li, 2013)
Total	85,598	0–7 Years	43,048/42,550	1.01	

The WHO Multicenter Growth Reference Study (MGRS) had a longitudinal design in its sample from birth to 24 months and cross-sectional design from 18 to 71 months. The CDC Growth Charts were established by longitudinal design, and the NSPGDC had a cross-sectional design. Similar to the fetal ultrasound measures, we calculated effect size measures within specific time epochs (per month). We used mean and standard deviation statistics from z-score tabulated data, which are openly available through the WHO, CDC, and NSPGDC (Kuczmarski et al., 2002; World Health Organization [WHO], 2007; Zong and Li, 2013). This included MGRS data from birth to 5 years, CDC Growth Chart data from birth to 3 years, and the full age range of the 4th National Survey on Physical Growth and Development of Children (NSPGDC-4) data, from birth to 7 years. Male-female ratios demonstrated relative parity in sex distribution. Across these three population surveillance databases 50th percentile head circumference (cm) values and standard deviation values are given for both males and females (Table 3).

### 2.2 Cortical morphometry

Thirty-three independent samples, from publicly accessible repositories, provided measures of cortical volume, surface area and thickness derived from whole brain T_1_-weighted MRI scans (Table 1). We used cross-sectional data and in the case of studies with longitudinal designs, we included only baseline data. We used individual-level data from each dataset selecting individuals who had high-quality MRI scans and without psychiatric, medical or neurological disorders at the time of scanning. Across all studies, the cortical measures examined were extracted from the individual MRI scans using standard pipelines implemented using the FreeSurfer image analysis suite v 7.^1^ For each participant in each dataset, FreeSurfer extracted measures of intracranial volume and bilateral total cortical surface area and total cortical thickness were used. Specifically, based on the Desikan–Killiany parcellation we used the summary fields SurfArea (mm^2^) and MeanThickness (mm); these correspond, respectively, to areal expansion and laminar thickness estimates computed after surface reconstruction and topology correction. Subsequently cortical volume in each participant was calculated as the product of surface area and thickness.

### 2.3 Calculation of Cohen’s d effect size

The Cohen’s d effect sizes were calculated using the following formula (McGrath and Meyer, 2006):


C⁢o⁢h⁢e⁢n′⁢s⁢d=x¯M-x¯FS⁢DM2+S⁢DF22

$\bar{x}$_*M*_ = Mean head circumference male

$\bar{x}$_*F*_ = Mean head circumference female

SD_*M*_ = Standard deviation male

SD_*F*_ = Standard deviation female

In prenatal papers 50th percentile ultrasound head circumference and standard deviation values were made available for both males and females (Table 3) by gestational week. In the population level datasets used in the assessment of head circumference in early childhood, as previously stated, 50th percentile head circumference and standard deviation values were made available for both males and females (Table 3) by month. Considering 50th percentile values as mean head circumference male and mean head circumference female, the ensuing calculation of Cohen’s d was a straightforward process, carried out for each month of available data. The exception was Melamed et al. (2013), in which these values were not available, and ultrasound mean head circumference and standard deviation were estimated through provided mean and standard deviation regression equations, by gestational week.

For the analyses evaluating effect sizes of MRI data across the lifespan, a sliding window algorithm was performed, programmed in R Studio (version 2023.09.0+463) and Python (version 3.12.7). With all 33 cross-sectional datasets concatenated into one large dataset, the algorithm detects the youngest and oldest age. The sliding window analysis then begins at the lower age of 4 years and creates a subset of data based on a window size of 4 years (all participants between 4 and 8 years-of-age). A sex-based effect size is then calculated for that specific window. The window then “slides” or increments by 2 years and the effect size calculation is repeated. This sliding window analysis performs sequential iterations across the complete age-range of the MRI data. The range of 4-years and step size of 2-years were chosen to balance the effects of data smoothing and resolution. The effect of individual datasets on sex differences is presented in Supplementary Figure 1.

### 2.4 Estimation of cortical volume and body-based effect size

Cortical volume for each individual with an MRI scan was calculated as the product of total surface area and cortical thickness. This estimated value was chosen preferentially over ICV since the development of the skull reaches a peak during early adolescence and remains stable into adulthood in healthy volunteers (Whitwell et al., 2001).

Analysis did not control for metrics of body size (height, weight, BMI, etc.). As such metrics were not available across all sources of data, analyses could not consistently control for body size.

## 3 Results

Within the prenatal, postnatal, and MRI periods, Cohen’s d effect size calculations of global sex differences were calculated and plotted relative to age. Factors that can influence Cohen’s d measures include mean difference (in the numerator) and pooled standard deviation (SD) (in the denominator). Thus, a larger Cohen’s d may be the result of a greater mean difference of brain measures between males and females, or alternatively, smaller pooled variance. Thus, we not only plotted the Cohen’s d measures, but also the mean difference and pooled SD for each measure.

### 3.1 Fetal measurements of head circumference

**FIGURE 1:**
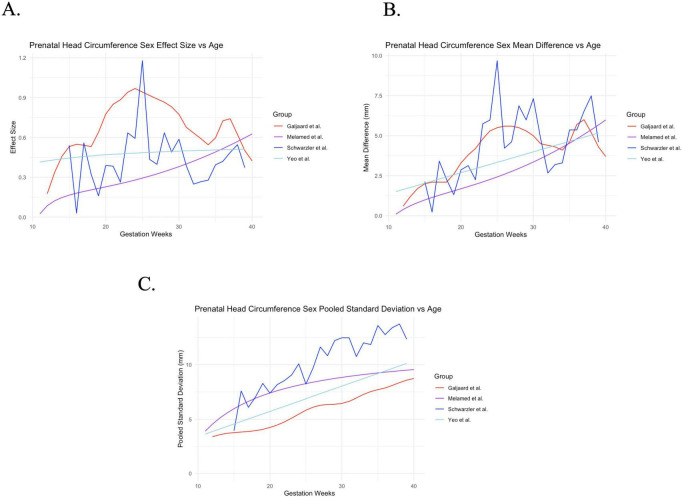
Prenatal results. **(A)** Prenatal head circumference effect size is plotted against gestational age. **(B)** Prenatal head circumference mean difference is plotted against gestational age. **(C)** Prenatal head circumference pooled standard deviation is plotted against gestational age.

Males consistently demonstrate a larger HC from the earliest data point (11 weeks gestational age). Head circumference effect size demonstrates a possible peak at 25 weeks, especially in the Galjaard et al. (2019) and Melamed et al. (2013) studies (Figure 1A). Yeo et al. (2017) demonstrates a near constant head circumference effect size around 0.45, while Melamed et al. (2013) demonstrates steady increase in effect size from 15 to 40 gestational weeks—these two prenatal datasets show smoother trends due to methodological differences in data smoothing.

Prenatal head circumference mean difference overall tracks closely with head circumference effect size results (Figure 1B), while the relationship between head circumference effect size and pooled standard deviation in general shows a gradual increase during fetal life (Figure 1C). It appears that average sex differences in head circumference drive head circumference effect size in these datasets, greater than variability in measurement—prenatal head circumference mean difference tracks closely with effect size.

Prenatal head circumference presents with substantial inconsistence between data sources (Figure 1A), likely a result of both smaller effect sizes and the cross-sectional design of most of the studies. Galjaard et al., having the largest sample size and a longitudinal design (Table 2) unsurprisingly shows the smoothest trend, which most closely resembles typical fetal development of head circumference and comparable 2D global brain measures such as brain fronto-occipital length and skull occipitofrontal diameter (Cai et al., 2020; Kyriakopoulou et al., 2017).

### 3.2 Direct head circumference measures in early childhood

**FIGURE 2:**
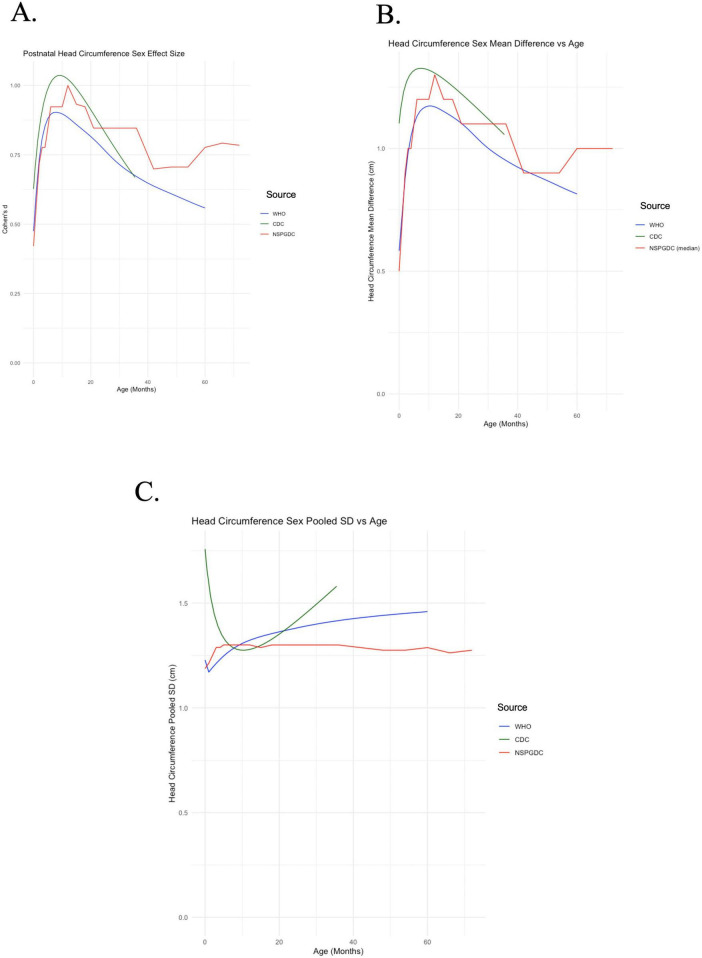
Postnatal results. **(A)** Postnatal head circumference effect size is plotted against age. **(B)** Postnatal head circumference mean difference is plotted against age. **(C)** Postnatal head circumference pooled standard deviation is plotted against age.

Postnatal head circumference effect size growth curves demonstrates that males consistently exhibit larger head circumference effect size from birth into early childhood (Figure 2A). The head circumference effect size shows a linear increase between birth until a peak between 8 and 10 months, which is followed by a steady decline between 10 and 60 months. There is a great degree of similarity between postnatal HC mean difference and head circumference effect size across early development (Figure 2B).

The CDC HC pooled standard deviation is markedly different than the WHO and NSPGDC data (Figure 2C). This may be related to the larger sample sizes or smoothing techniques utilized in the WHO and NSPGDC growth curves, or perhaps different factors of population diversity (nutritional, genetic and ancestral background, socioeconomic, etc.) in the US-based CDC dataset. Similar to the effect sizes seen during fetal life, average sex differences appear to play a larger role in driving postnatal head circumference effect size than a decrease in the pooled SD.

### 3.3 MRI results

**FIGURE 3:**
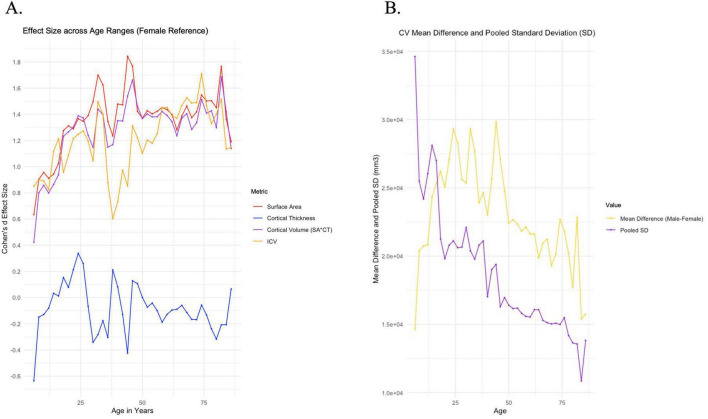
MRI results. **(A)** Surface area, cortical thickness, cortical volume, and intracranial volume (ICV) effect size is plotted against age range. **(B)** CV mean difference is plotted against age range (from sliding window analysis). CV pooled standard deviation is plotted against age range.

Males show greater surface area and cortical volumes across the entire timeline (Figure 3A). Effect sizes for both surface area and cortical volume show a steady increase from a Cohen’s d of approximately 0.4 at 5 years-of-age to a Cohen’s d of approximately 1.4 at 24 years-of-age, after which the Cohen’s d remains relatively stable. The effect size measure of cortical thickness, however, is negative (females greater than males) during childhood, but approaches zero at approximately 13 years-of-age.

MRI cortical volume mean difference and pooled standard deviation were reported in Figure 3B to demonstrate the relative contributions of the numerator and denominator in the Cohen’s d effect size formula. The numerator reflects the mean difference of cortical volume between males and females and the denominator reflects the pooled standard deviation. MRI cortical volume mean difference tracks well with CVES, while pooled standard deviation follows roughly an inverse pattern of mean difference (Figure 3B). Such trends suggest that mean difference and variability in measurement both contribute to differences in CVES across the MRI period. Lifespan data points seen over age 74 are informed by low sample size (*n* < 60) and are therefore considered to be of lower confidence. Data points generally have varying confidence, which can be assessed through Supplementary Figure 2.

### 3.4 Combined results

**FIGURE 4:**
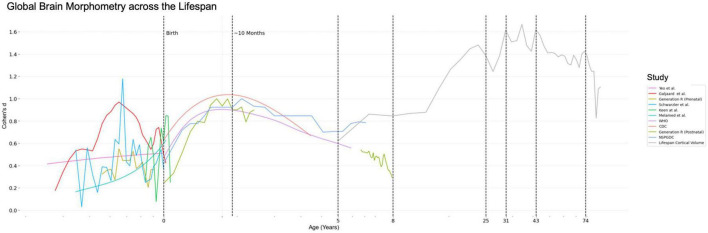
Lifespan results. Head circumference effect size and cortical volume effect size are plotted against gestational age in the prenatal period, age in the postnatal period, and age range in the MRI period. A log scale is utilized, ages are demarcated, and this figure comprises a combination of Figures 1–3 and represents a goal of the research project—to map morphometric effect size across the lifespan.

For visualization from prenatal life into old age, effect size computations were combined across all modalities (Figure 4). Combined results compare prenatal HCES, postnatal HCES, and cortical volume effect size (CVES). Despite variability introduced by varied sample sizes, cohort effects, and data collection methods, head circumference/cortical volume is consistently greater in males than females (Figure 4). Prenatal HCES demonstrates a greater level of variability. Rapid postnatal HCES growth is observed between birth and 10 months of age and CVES growth between ages 15–19. CVES is quite stable after age 25. Interestingly, while CVES does not vary notably after age 25, the peak HCES or CVES effect size values are observed in this age range.

## 4 Discussion

### 4.1 Major findings

The aim of this study was to assess sex-based effect sizes of global brain measures across the lifespan, from uterine life to old age. In our analysis of 147,931 subjects between 11 weeks gestational age and 89 years, several primary findings emerged. First, we found that males had larger measures of brain volume as inferred from head circumference, beginning as early as the 11th week of prenatal life. However, the Cohen’s d measure was not constant over the lifespan. The Cohen’s d measure had a potential early peak between 20 and 25 weeks gestational age, a second peak at approximately 10 months postnatal, which was followed by a decline until late childhood. Interestingly, within the prenatal period (0–7 years), population-level growth chart data from the WHO, CDC, and NSPGDC all cohered to show a peak in effect size around 10 months of age. Finally, based on the MRI data, there is a subsequent increase in brain size beginning in mid-childhood until young adulthood. This time window is synonymous with pubertal development. There was an increase in the Cohen’s d measure until it reached a plateau of approximately 1.4 at approximately 25 years-of-age, with evidence for a gradual decline after the age of 40 years.

### 4.2 Embedding with existing literature

In Ruigrok et al. (2014) meta-analysis of 7,515 participants across 57 studies with mean ages ranging from 4 to 70 years, a fixed-effects (FFX) analysis revealed a large effect size favoring males for total gray matter volume, with a Cohen’s d of 2.13 (Ruigrok et al., 2014). While higher than the CVES Cohen’s d in our analysis, in which we showed a mean MRI CVES between 4 and 70 years of approximately 1.25, this difference may be attributable to cohort effects or differences between our measure of cortical volume and their measure of total gray matter volume, which includes subcortical gray matter volume. Further, a Cohen’s d of 2.13 is very large and using linear classifiers, equates to a 93.3% classification accuracy, which appears overly optimistic. We found a gradual decline in CVES after age 43, which was driven largely by the UK Biobank study (*n* > 1,500). This decline in overall CVES may explain the discrepancy related to the GM FFX finding, which largely focused on populations in middle adulthood or younger. Nevertheless, we did not show a CVES > 1.6 at any time.

We found a lower global sex effect size on volumetric assessment of cerebral cortex compared to Ruigrok et al.’s (2014) analysis. While our methodological approach was different from Ruigrok et al. (2014) we had a larger sample size and this may more closely approximate population differences. The meta-analysis also confirms the global effect trend of M > F across a large number of volume measures, intracranial volume (3.03), total brain volume (2.1), cerebrum volume (3.35), gray matter (2.13), white matter (2.06), cerebrospinal fluid (1.21), and cerebellar volume (1.68). Broadly speaking, our work supports pre-existing literature on volumetric global effect sizes between males and females, only perhaps raising the possibility of such effects are larger compared to population-level differences. Further, the meta-analysis assessed effect size measures over the entire age spectrum and did not assess age-related differences. Importantly, while a number of studies indicate that global brain volume measures themselves (total brain volume) vary substantially by chronological age (Bethlehem et al., 2022), to the best of our knowledge, no studies have effectively examined the relationship between sex-based global brain effect sizes at different ages.

Various findings are reported in existing literature on the relationship between gray matter volume, age and sex. Gennatas et al. (2017) demonstrated in the 1,189 children from the Philadelphia Neurodevelopmental Cohort roughly parallel decreases in gray matter volume for males and females between 8 and 23 years of age. Using the UK Biobank, Ritchie et al. (2018) found the distributions for both total and regional gray matter volumes, including both cortical and subcortical regions, demonstrated to be shifted rightward and to be wider in males compared to females, indicating higher means and greater variance in males. Bethlehem et al. (2022) aggregated 123,984 MRI scans from more than 100 studies covering mid-gestation to 100 postnatal years and used GAMLSS modeling to yield normative trajectories of gray matter volume. The growth curves between males and females are largely parallel, with the greatest differentiation seen roughly between the ages of 4–16 years.

What these studies did not assess was the subtle differences between the sexes in growth trajectories. When plotting growth curves by sex using means and standard deviations, the gross differences are visible, however, the clear peaks seen during prenatal, late in the first year of life, and surrounding puberty become readily apparent when assessing effect size differences.”

Our analysis largely compliments the above findings, demonstrating relatively small yet increasing cortical volume sex effect size from around 0.4 around 5–6 years of age to 1.4 around 23–24 years of age, and dropping beneath 1.4 in a more stable trend later in life. Small effects are consistent with trajectories of gray matter observed to be parallel and close in proximity between males and females throughout much of development, while increasing effect size into adolescence is consistent with larger gray matter differentiation seen in Bethlehem et al. (2022) between 4 and 16 years. Small prenatal head circumference effect size (generally near 0–0.6, as seen in Figure 1) and early childhood (generally near 0.5–1, as seen in Figure 2) additionally adds support to literature which shows gray matter trajectories not dramatically differentiated between males and females.

### 4.3 Novel findings

While we do not have biological samples obtained at different time points over the lifespan, one hypothesis is that underlying developmental neuroendocrine processes between the sexes are related to the observed variations in effect size over time. Males typically have three periods associated with increased secretion of testosterone; the first beginning during early prenatal life, the second in early postnatal life, and the third taking place beginning in adolescence (Hines et al., 2016; Nassar and Leslie, 2018; Rey et al., 2020). The initial surge of testosterone in males begins around 8 weeks gestational age and is associated with the development of the primary sexual organ development (Hines et al., 2015; Rey et al., 2020). This surge could be responsible for the first peak in effect size during prenatal life. It has been well documented that a “mini-puberty” testosterone surge occurs between 1 and 6 months postnatally in males (Hines et al., 2016; Kuiri-Hänninen et al., 2014). This early testosterone surge may be associated with the initial separation and growth in male head circumference compared to females. This surge could influence cortical growth in males over females and account for the peak Cohen’s d between 8 and 10 months of age. The final peak begins at the time of puberty, a period of time in which we observed a gradual increase in the Cohen’s d measure up to the age of 25 years. This also could be either directly or indirectly related to the large increase in testosterone in males compared to females. However, what is also interesting is the decrease in Cohen’s d after 10 months of age up to the time of puberty, which suggests the 10-month growth spurt is followed by a slower growth rate in relation to females.

Further, it is possible that a connection may be drawn between increase in CVES from 5 to 25 years and neurodevelopmental processes which have been known to proceed throughout early adulthood. Such processes include myelination and synaptic pruning assessed via dendritic spine density (Miller et al., 2012), and executive function (Petanjek et al., 2011). In any case, it is essential to regard connections between neurodevelopment and morphometric change as purely speculative. New studies that systematically evaluate endocrine, (epi)genetic and neuroanatomical aspects of development (incorporating blood-based neurodevelopmental biomarkers) are necessary to empirically defend an underlying mechanism of morphometry effect size changes.

It is interesting that cortical thickness effect size was negative (F > M) from early childhood into early adolescence and pertinent to consider differences in pubertal timing. This finding may be related to the differing time course of sexual maturation and production of gonadal hormones in females. Previous literature has observed associations between volumetric gray matter sex differences and circulating levels of sex hormones or androgen receptor genotype (Paus et al., 2010; Pletzer, 2019; Witte et al., 2010). Such differences might not be seen in a sample matched on pubertal markers, such as freely circulating testosterone rather than analyzed using chronological age, which has been described in literature concerning cortical thickness (Bramen et al., 2011).

A number of studies have explored the relationship between puberty, endocrinology, and brain morphology (Bramen et al., 2012; Herting et al., 2015; Vijayakumar et al., 2021). Such work identifies noteworthy associations and interactions between sex, age, circulating testosterone, and both widespread and regional cortical thickness and cortical surface area. It is interesting to observe these findings in the context of our finding greater cortical thickness in girls in early adolescence. It is essential to acknowledge the multifactorial landscape that surrounds any tentative relationship between endocrinology and brain development. Gene regulation, testosterone, estrogen, progesterone, adrenal androgens, gonadotropins, growth hormones, and other factors all interact with one another and with the development of the brain in puberty and beyond (Herting and Sowell, 2017; Sisk and Zehr, 2005; Sisk, 2017).

### 4.4 Strengths and limitations

This work provides a unique contribution to the field of brain morphometry and neurodevelopmental sex differences. While sex differences have been examined in a large range of studies across the age range, analyses have not evaluated age-related differences between the sexes, which is the major strength of our study. An additional strength of our study is the large sample size (total *n* = 147,931) spanning from prenatal life to old age. Simultaneously, though, achieving such a large sample size required the combination of data from a large variety of studies with inconsistent sample sizes, demographic features, and experimental designs. This is especially the case with respect to the MRI data, which includes structural MRI data from 33 independent datasets. Other limitations include that we were unable to assess the sex-based effect size differences related to body size with the MRI data, and that trends observed above age 70 generally had smaller contributing sample size and thus lower confidence. It is a limitation of the paper that the same brain metrics were not used across all analyses, and that data collected via MRI were done using multiple different scanners. However, global brain measures are have high reliability between different MR platforms. Finally, we used different global brain measures between the prenatal/postnatal (head circumference) and MRI (cortical metrics) periods.

For the MRI component of the study, having raw data from 33 different studies with each study using different scanners and different sequences creates challenges in data harmonization. When we accounted for ICV there continued to be considerable variability, which suggests that accounting for global metrics will not remove all variability. The data that we used has undergone quality control and removal of poor-quality data as presented in the origin publication (Frangou et al., 2022; Ge et al., 2024; Yu et al., 2024). While there are methods that we could have applied to account for the different sites, and thus scanner and sequence differences, there was concern that these approaches could alter age-related differences. Imaging can have inherent noise and we decided to present the findings with the noise, especially since the underlying patterns are readily identified in spite of the noise.

The utilization of head circumference metrics in the calculation of prenatal and postnatal effect sizes presupposes that head circumference effectively represents a “global brain” metric. In other words, we presuppose that head circumference can be said to strongly predict other more direct measures of the global brain, such as intracranial volume or total brain volume. Head circumference has been demonstrated to predict total brain volume well within neonates (Lindley et al., 1999), and healthy children 1.7–6 years old (Bartholomeusz et al., 2002). These findings lend general support to the presupposition that head circumference can be considered a representative measure of the global brain in our age range of interest, the middle prenatal period to 7 years old.

All observed samples were population-based samples or composed of typically developing volunteers, rather than being clinical studies. Still, it is possible that in population-based studies quantifying either pre- or postnatal head circumference could entail individuals with conditions that would skew the findings (e.g., enlarged ventricles or ASD diagnoses). Measures of psychopathology and ventricular size in these datasets was unavailable. It should be acknowledged that the MRI dataset contains data from a variety of 1.5 and 3T MRI scanners, suggesting a variety in image quality. Under- and over segmentation effects introduce a possible source of noise into sex-based effect size findings.

It is worth mentioning the distinctively different curves between the WHO and NSPGDC pooled standard deviation trends and that trend observed in the CDC dataset (Figure 2C), and the general tendency for pooled standard deviation to change over the prenatal period. “The age-related differences in the sex pooled SD between the CDC and the WHO and NSPGDC (Figure 2C) are perplexing, however, this may relate to differences in how the data was processed between the studies. HC measures performed at or closely after birth, a time when the skull passes through the birth canal, have much greater standard deviations than measures taken a month later. Thus, one possibility is that a measure at the time of birth in the CDC sample, coupled with a smoothing algorithm that blurs this variability to later timepoints, could result in a greater pooled SD within the first several months after birth. As mentioned previously in 3.2, pooled standard deviation trends are affected both by methodological differences in data smoothing and sample size, and general factors of population diversity (nutrition, genetic and ancestral background, socioeconomic status, etc.).

### 4.5 Implications for machine learning

There have been a number of studies using structural brain MRI metrics and machine learning algorithms to predict the sex of an individual (Bi et al., 2023; Mendes et al., 2021; Sepehrband et al., 2018). Our work suggests that the age of the participants will influence the contribution of the global brain measures in predictive algorithms. Specifically, our work suggests a peak head circumference effect size at 10 months-of-age followed by a large decline, increasing CVES between 5 and 25 years, and large, stable CVES beyond 25 years. Such findings indicate that training models on structural MRI data of participants in adults may yield more accurate classifications of sex due to the greater contribution of global brain differences. This is interesting, as much of the work in this area has been focused in studies with an average participant age of 15 years or younger (Bi et al., 2023; Mendes et al., 2021; Sepehrband et al., 2018).

### 4.6 Clinical implications

There are marked sex differences in both the prevalence and average age of onset in a large variety of neurodevelopmental and psychiatric disorders, including autism spectrum disorder (Ferri et al., 2018; McDonnell et al., 2021), schizophrenia (Abel et al., 2010), major depressive disorder (Essau et al., 2010), and several anxiety disorders (Farhane-Medina et al., 2022), to name a few. A number of disorders with a clinical profile encompassing internalizing symptoms (e.g., MDD and generalized anxiety) frequently demonstrate higher rates and earlier onset in females (Ellis et al., 2017; McLaughlin and King, 2015; Ohannessian et al., 2017). Our work may serve to indicate underlying periods of brain development in which sex-based vulnerability to specific psychiatric disorders is highest. This applies to anxious and depressive symptomatology, but also may more broadly be applicable to a range of mental health disorders with sex-based vulnerability.

The relationship between sex/sex-specific brain morphology and mental disorders, it certainly should be noted, interacts with a great variety of third variables. Several sociological correlates of sex in gender, gender roles, and gender-specific environmental stressors (Brummett et al., 2008; Christiansen et al., 2022; Howard et al., 2017) stand to influence the relationship between sex and mental health outcomes, along with other biological factors such as sex-linked genetic predisposition, peripartum neurobiology, and menopause (Cárdenas et al., 2020; Ngun et al., 2011; Than et al., 2021). When considering clinical implications of sex differences on mental health outcomes, the discussion is definitionally multifactorial.

Our analysis appears to have relevance to the discussion of “sexual dimorphisms” in brain morphometry. To be precise, “sexual dimorphisms” in historical scientific discourse have been most recognized in animal neurobiology research—specific, delimited structures or brain circuits that differ structurally, disproportionately, and often dramatically between males and females and underlie sex-specific behaviors such as courtship or mating (Breedlove and Arnold, 1980; Eliot et al., 2021; Gorski et al., 1978; Nottebohm and Arnold, 1976). Some have argued that this technical definition of “dimorphism” has failed to yield substantive parallels in human studies based on structural MRI (Eliot et al., 2021). In meta-analysis data spanning three decades of structural MR-based human neuroimaging studies, few broad male/female differences survive correction for total brain volume and lateralization, with sex explaining approximately 1% of the total variance after correction (Eliot et al., 2021). Relevant proposed regional sex-differences address findings of higher white/gray matter ratio, intra- vs. interhemispheric connectivity, and both cortical and subcortical volumes.

This is not to admit a total absence of sexually dimorphic regions, with one study, for example, demonstrating distinct neuronal histological differences in the bed nucleus of the stria terminalis (BNST) —the stria terminalis being a long, curved white matter fiber tract extending from the amygdala toward anterior hypothalamus (Bao and Swaab, 2010; Naidich et al., 2013; Wycoco et al., 2013). The BNST is implicated in limbic interaction, amygdalar output, and gonadotropin secretion via the hypothalamic adrenal axis (Handa and Weiser, 2014; Mbiydzenyuy and Qulu, 2024) —autonomic functions which, across various mammalian animal models and human studies, have demonstrated notable sex-specific profiles and which are related to sex-specific behavioral differences in emotional regulation, stress, fear, aggressive behavior, and even sexual behavior (Goel et al., 2014; Heinrichs et al., 1997; Mbiydzenyuy and Qulu, 2024). A number of other papers have found noteworthy morphometric and neurological sex differences between males and females in the BNST (Allen and Gorski, 1990; Allen et al., 1989). Neurological sex differences have additionally been observed in the BNST of rodents and non-human primates in stress paradigms (Urien and Bauer, 2022; Wright et al., 2023).

It is known that global brain measures (e.g., ICV, TBC, GMV, WMV) vary, and vary dramatically between males and females (Dean et al., 2018; Knickmeyer et al., 2017; Ruigrok et al., 2014). These global differences do not fit clearly within the definition of “dimorphism,” as a *categorical*, behaviorally-linked differences. However, these continuous differences remain relevant to a fundamental neurological understanding of sex differences and, as discussed previously, to the potential for clinical understanding and prediction.

### 4.7 Future directions

Our study serves as an exploration of global brain sex differences at different ages across the lifespan. Future work could integrate brain relative to body size, limit studies with smaller sample sizes, integrate demographic variables, or incorporate purely longitudinal data. Further, integrating studies with physiological events, including the collection of biosamples, may help identify factors driving the age-related sex differences. This is especially true of the distinctive peak in head circumference effect size observed around 10 months of age, which interestingly may correspond to increased cerebral growth in a subset of youth with autism spectrum disorder (Lee et al., 2021). Indeed, the most meaningful application of this work is to extend this line of research to clinical populations (e.g., autism, generalized anxiety, MDD).

## 5 Conclusion

We found a positive Cohen’s d effect size, M > F in global brain measures beginning in prenatal life and extending throughout the lifespan. There is evidence for a possible peak in effect size at approximately 20–25 weeks gestational age, although this was not seen in all studies. However, there was a consistent peak in head circumference effect size at approximately 10 months-of-age with Cohen’s d of approximately 1.1 (Figure 2). This peak is seen consistently across four large population-based head circumference datasets. CVES appears to show an almost linear increase beginning in childhood and increasing to the age of 25 years. Additionally, the cortical thickness effect size shows a period in which females are greater than males prior to middle adolescence (Figure 3). Overall, we found that sex differences were seen to vary by chronological age. Such findings have interesting potential explanations related to active endocrine and neurodevelopmental processes in certain age windows, notably during early postnatal life and adolescence into early adulthood. In addition, machine-learning prediction models used to predict sex will be influenced by the age of the participants. Our analysis, in combination with future follow-up studies, could prove relevant to clinical disorders which vary in prevalence and age of onset by sex.

## Data Availability

The data analyzed in this study is subject to the following licenses/restrictions: We have provided all links to existing data and for those datasets that are not freely available, it is possible to contact the individual sites and work with them via a data transfer agreement between institutions. All code used in the analyses is made publicly available in the following GitHub repository: https://github.com/SoCoDeN/dimorphism. Prenatal and head circumference data were extracted from existing publications. This was a multi-site study and the information is contained in the manuscript (see Table 1).

## Footnotes

1
https://freesurfer.net/

## Author disclaimer

This research was supported by the Intramural Research Program of the National Institutes of Health (NIH). The contributions of the NIH author(s) are considered Works of the United States Government. The findings and conclusions presented in this paper are those of the author(s) and do not necessarily reflect the views of the NIH or the U.S. Department of Health and Human Services.

## References

[B1] Abel K. M. Drake R. Goldstein J. M. (2010). Sex differences in schizophrenia. *Int. Rev. Psychiatry* 22 417–428. 10.3109/09540261.2010.515205 21047156

[B2] Allen L. S. Gorski R. A. (1990). Sex difference in the bed nucleus of the stria terminalis of the human brain. *J. Comp. Neurol.* 302 697–706. 10.1002/cne.903020402 1707064

[B3] Allen L. S. Hines M. Shryne J. E. Gorski R. A. (1989). Two sexually dimorphic cell groups in the human brain. *J. Neurosci.* 9 497–506. 10.1523/JNEUROSCI.09-02-00497.1989 2918374 PMC6569815

[B4] Bao A. M. Swaab D. F. (2010). Sex differences in the brain, behavior, and neuropsychiatric disorders. *Neuroscientist* 16 550–565. 10.1177/1073858410377005 20889965

[B5] Bartholomeusz H. H. Courchesne E. Karns C. M. (2002). Relationship between head circumference and brain volume in healthy normal toddlers, children, and adults. *Neuropediatrics* 33 239–241. 10.1055/s-2002-36735 12536365

[B6] Bethlehem R. A. I. Seidlitz J. White S. R. Vogel J. W. Anderson K. M. Adamson C. (2022). Brain charts for the human lifespan. *Nature* 604 525–533. 10.1038/s41586-022-04554-y 35388223 PMC9021021

[B7] Bi Y. Abrol A. Fu Z. Chen J. Liu J. Calhoun V. (2023). Prediction of gender from longitudinal MRI data via deep learning on adolescent data reveals unique patterns associated with brain structure and change over a two-year period. *J. Neurosci. Methods* 384:109744. 10.1016/j.jneumeth.2022.109744 36400261

[B8] Blanken L. M. E. Dass A. Alvares G. van der Ende J. Schoemaker N. K. El Marroun H. (2018). A prospective study of fetal head growth, autistic traits and autism spectrum disorder. *Autism Res.* 11 602–612. 10.1002/aur.1921 29356450 PMC5947578

[B9] Blanken L. M. Mous S. E. Ghassabian A. Muetzel R. L. Schoemaker N. K. El Marroun H. (2015). Cortical morphology in 6- to 10-year old children with autistic traits: A population-based neuroimaging study. *Am. J. Psychiatry* 172 479–486. 10.1176/appi.ajp.2014.14040482 25585034

[B10] Bölte S. Neufeld J. Marschik P. B. Williams Z. J. Gallagher L. Lai M. C. (2023). Sex and gender in neurodevelopmental conditions. *Nat. Rev. Neurol.* 19 136–159. 10.1038/s41582-023-00774-6 36747038 PMC10154737

[B11] Bracké K. F. M. Steegers C. P. M. van der Harst T. Dremmen M. H. G. Vernooij M. W. White T. J. H. (2023). Can neuroimaging measures differentiate the disease course of anorexia nervosa? A systematic review. *J. Psychiatr. Res.* 163 337–349. 10.1016/j.jpsychires.2023.05.059 37263169

[B12] Bramen J. E. Hranilovich J. A. Dahl R. E. Chen J. Rosso C. Forbes E. E. (2012). Sex matters during adolescence: Testosterone-related cortical thickness maturation differs between boys and girls. *PLoS One* 7:e33850. 10.1371/journal.pone.0033850 22479458 PMC3315517

[B13] Bramen J. E. Hranilovich J. A. Dahl R. E. Forbes E. E. Chen J. Toga A. W. (2011). Puberty influences medial temporal lobe and cortical gray matter maturation differently in boys than girls matched for sexual maturity. *Cereb. Cortex* 21 636–646. 10.1093/cercor/bhq137 20713504 PMC3041011

[B14] Breedlove S. M. Arnold A. P. (1980). Hormone accumulation in a sexually dimorphic motor nucleus of the rat spinal cord. *Science* 210 564–566. 10.1126/science.7423210 7423210

[B15] Brummett B. H. Boyle S. H. Siegler I. C. Kuhn C. M. Ashley-Koch A. Jonassaint C. R. (2008). Effects of environmental stress and gender on associations among symptoms of depression and the serotonin transporter gene linked polymorphic region (5-HTTLPR). *Behav. Genet.* 38 34–43. 10.1007/s10519-007-9172-1 17955359 PMC2777886

[B16] Cai S. Zhang G. Zhang H. Wang J. (2020). Normative linear and volumetric biometric measurements of fetal brain development in magnetic resonance imaging. *Childs Nerv. Syst.* 36 2997–3005. 10.1007/s00381-020-04633-3 32468242

[B17] Cárdenas E. F. Kujawa A. Humphreys K. L. (2020). Neurobiological changes during the peripartum period: Implications for health and behavior. *Soc. Cogn. Affect. Neurosci.* 15 1097–1110. 10.1093/scan/nsz091 31820795 PMC7657461

[B18] Christiansen D. M. McCarthy M. M. Seeman M. V. (2022). Where sex meets gender: How sex and gender come together to cause sex differences in mental illness. *Front. Psychiatry* 13:856436. 10.3389/fpsyt.2022.856436 35836659 PMC9273892

[B19] Dean D. C. Planalp E. M. Wooten W. Schmidt C. K. Kecskemeti S. R. Frye C. (2018). Investigation of brain structure in the 1-month infant. *Brain Struct. Funct.* 223 1953–1970. 10.1007/s00429-017-1600-2 29305647 PMC5886836

[B20] Eaton N. R. Keyes K. M. Krueger R. F. Balsis S. Skodol A. E. Markon K. E. (2012). An invariant dimensional liability model of gender differences in mental disorder prevalence: Evidence from a national sample. *J. Abnorm. Psychol.* 121 282–288. 10.1037/a0024780 21842958 PMC3402021

[B21] Eliot L. Ahmed A. Khan H. Patel J. (2021). Dump the “dimorphism”: Comprehensive synthesis of human brain studies reveals few male-female differences beyond size. *Neurosci. Biobehav. Rev.* 125 667–697. 10.1016/j.neubiorev.2021.02.026 33621637

[B22] Ellis R. E. R. Seal M. L. Simmons J. G. Whittle S. Schwartz O. S. Byrne M. L. (2017). Longitudinal trajectories of depression symptoms in adolescence: Psychosocial risk factors and outcomes. *Child Psychiatry Hum. Dev.* 48 554–571. 10.1007/s10578-016-0682-z 27619221

[B23] Essau C. A. Lewinsohn P. M. Seeley J. R. Sasagawa S. (2010). Gender differences in the developmental course of depression. *J. Affect. Disord.* 127 185–190. 10.1016/j.jad.2010.05.016 20573404 PMC3754427

[B24] Farhane-Medina N. Z. Luque B. Tabernero C. Castillo-Mayén R. (2022). Factors associated with gender and sex differences in anxiety prevalence and comorbidity: A systematic review. *Sci. Prog.* 105:368504221135469. 10.1177/00368504221135469 36373774 PMC10450496

[B25] Ferri S. L. Abel T. Brodkin E. S. (2018). Sex differences in autism spectrum disorder: A review. *Curr. Psychiatry Rep.* 20:9. 10.1007/s11920-018-0874-2 29504047 PMC6477922

[B26] Frangou S. Modabbernia A. Williams S. C. R. Papachristou E. Doucet G. E. Agartz I. (2022). Cortical thickness across the lifespan: Data from 17,075 healthy individuals aged 3-90 years. *Hum. Brain Mapp.* 43 431–451. 10.1002/hbm.25364 33595143 PMC8675431

[B27] Gafner M. Fried S. Gosher N. Jeddah D. Sade E. K. Barzilay E. (2020). Fetal brain biometry: Is there an agreement among ultrasound, MRI and the measurements at birth? *Eur. J. Radiol.* 133:109369. 10.1016/j.ejrad.2020.109369 33126174

[B28] Galjaard S. Ameye L. Lees C. C. Pexsters A. Bourne T. Timmerman D. (2019). Sex differences in fetal growth and immediate birth outcomes in a low-risk Caucasian population. *Biol. Sex Differ.* 10:48. 10.1186/s13293-019-0261-7 31500671 PMC6734449

[B29] Ge R. Yu Y. Qi Y. X. Fan Y. N. Chen S. Gao C. (2024). Normative modelling of brain morphometry across the lifespan with CentileBrain: Algorithm benchmarking and model optimisation. *Lancet Digit. Health* 6 e211–e221. 10.1016/S2589-7500(23)00250-9 38395541 PMC10929064

[B30] Gennatas E. D. Avants B. B. Wolf D. H. Satterthwaite T. D. Ruparel K. Ciric R. (2017). Age-related effects and sex differences in gray matter density, volume, mass, and cortical thickness from childhood to young adulthood. *J. Neurosci.* 37 5065–5073. 10.1523/JNEUROSCI.3550-16.2017 28432144 PMC5444192

[B31] Goel N. Workman J. L. Lee T. T. Innala L. Viau V. (2014). Sex differences in the HPA axis. *Compr. Physiol.* 4 1121–1155. 10.1002/cphy.c130054 24944032

[B32] Gorski R. A. Gordon J. H. Shryne J. E. Southam A. M. (1978). Evidence for a morphological sex difference within the medial preoptic area of the rat brain. *Brain Res.* 148 333–346. 10.1016/0006-8993(78)90723-0 656937

[B33] Handa R. J. Weiser M. J. (2014). Gonadal steroid hormones and the hypothalamo-pituitary-adrenal axis. *Front. Neuroendocrinol.* 35 197–220. 10.1016/j.yfrne.2013.11.001 24246855 PMC5802971

[B34] Heinrichs S. C. Min H. Tamraz S. Carmouché M. Boehme S. A. Vale W. W. (1997). Anti-sexual and anxiogenic behavioral consequences of corticotropin-releasing factor overexpression are centrally mediated. *Psychoneuroendocrinology* 22 215–224. 10.1016/s0306-4530(97)00030-9 9226726

[B35] Herting M. M. Sowell E. R. (2017). Puberty and structural brain development in humans. *Front. Neuroendocrinol.* 44 122–137. 10.1016/j.yfrne.2016.12.003 28007528 PMC5612369

[B36] Herting M. M. Gautam P. Spielberg J. M. Dahl R. E. Sowell E. R. (2015). A longitudinal study: Changes in cortical thickness and surface area during pubertal maturation. *PLoS One* 10:e0119774. 10.1371/journal.pone.0119774 25793383 PMC4368209

[B37] Hines M. Constantinescu M. Spencer D. (2015). Early androgen exposure and human gender development. *Biol. Sex Differ.* 6:3. 10.1186/s13293-015-0022-1 25745554 PMC4350266

[B38] Hines M. Spencer D. Kung K. T. Browne W. V. Constantinescu M. Noorderhaven R. M. (2016). The early postnatal period, mini-puberty, provides a window on the role of testosterone in human neurobehavioural development. *Curr. Opin. Neurobiol.* 38 69–73. 10.1016/j.conb.2016.02.008 26972372

[B39] Hoogman M. Muetzel R. Guimaraes J. P. Shumskaya E. Mennes M. Zwiers M. P. (2019). Brain imaging of the cortex in ADHD: A coordinated analysis of large-scale clinical and population-based samples. *Am. J. Psychiatry* 176 531–542. 10.1176/appi.ajp.2019.18091033 31014101 PMC6879185

[B40] Howard L. M. Ehrlich A. M. Gamlen F. Oram S. (2017). Gender-neutral mental health research is sex and gender biased. *Lancet Psychiatry* 4 9–11. 10.1016/S2215-0366(16)30209-7 27856394

[B41] Hshieh T. T. Fox M. L. Kosar C. M. Cavallari M. Guttmann C. R. Alsop D. (2016). Head circumference as a useful surrogate for intracranial volume in older adults. *Int. Psychogeriatr.* 28 157–162. 10.1017/S104161021500037X 26631180 PMC4669896

[B42] Jäncke L. Mérillat S. Liem F. Hänggi J. (2015). Brain size, sex, and the aging brain. *Hum. Brain Mapp.* 36 150–169. 10.1002/hbm.22619 25161056 PMC6869393

[B43] Keen D. V. Pearse R. G. (1988). Weight, length, and head circumference curves for boys and girls of between 20 and 42 weeks’ gestation. *Arch. Dis. Child.* 63 1170–1172. 10.1136/adc.63.10_spec_no.1170 3196071 PMC1590216

[B44] Knickmeyer R. C. Xia K. Lu Z. Ahn M. Jha S. C. Zou F. (2017). Impact of demographic and obstetric factors on infant brain volumes: A population neuroscience study. *Cereb. Cortex* 27 5616–5625. 10.1093/cercor/bhw331 27797836 PMC6075568

[B45] Kuczmarski R. J. Ogden C. L. Guo S. S. Grummer-Strawn L. M. Flegal K. M. Mei Z. (2002). 2000 CDC Growth Charts for the United States: Methods and development. *Vital Health Stat. 11* 246 1–190. 12043359

[B46] Kuiri-Hänninen T. Sankilampi U. Dunkel L. (2014). Activation of the hypothalamic-pituitary-gonadal axis in infancy: Minipuberty. *Horm. Res. Paediatr.* 82 73–80. 10.1159/000362414 25012863

[B47] Kyriakopoulou V. Vatansever D. Davidson A. Patkee P. Elkommos S. Chew A. (2017). Normative biometry of the fetal brain using magnetic resonance imaging. *Brain Struct. Funct.* 222 2295–2307. 10.1007/s00429-016-1342-6 27885428 PMC5504265

[B48] Lee J. K. Andrews D. S. Ozonoff S. Solomon M. Rogers S. Amaral D. G. (2021). Longitudinal evaluation of cerebral growth across childhood in boys and girls with autism spectrum disorder. *Biol. Psychiatry* 90 286–294. 10.1016/j.biopsych.2020.10.014 33388135 PMC8089123

[B49] Lenroot R. K. Gogtay N. Greenstein D. K. Wells E. M. Wallace G. L. Clasen L. S. (2007). Sexual dimorphism of brain developmental trajectories during childhood and adolescence. *Neuroimage* 36 1065–1073. 10.1016/j.neuroimage.2007.03.053 17513132 PMC2040300

[B50] Lindley A. A. Benson J. E. Grimes C. Cole T. M. Herman A. A. (1999). The relationship in neonates between clinically measured head circumference and brain volume estimated from head CT-scans. *Early Hum. Dev.* 56 17–29. 10.1016/s0378-3782(99)00033-x 10530903

[B51] Lüders E. Steinmetz H. Jäncke L. (2002). Brain size and grey matter volume in the healthy human brain. *Neuroreport* 13 2371–2374. 12488829

[B52] Matthew J. Malamateniou C. Knight C. L. Baruteau K. P. Fletcher T. Davidson A. (2018). A comparison of ultrasound with magnetic resonance imaging in the assessment of fetal biometry and weight in the second trimester of pregnancy: An observer agreement and variability study. *Ultrasound* 26 229–244. 10.1177/1742271X17753738 30479638 PMC6243456

[B53] Mbiydzenyuy N. E. Qulu L. A. (2024). Stress, hypothalamic-pituitary-adrenal axis, hypothalamic-pituitary-gonadal axis, and aggression. *Metab. Brain Dis.* 39 1613–1636. 10.1007/s11011-024-01393-w 39083184 PMC11535056

[B54] McDonnell C. G. DeLucia E. A. Hayden E. P. Penner M. Curcin K. Anagnostou E. (2021). Sex differences in age of diagnosis and first concern among children with autism spectrum disorder. *J. Clin. Child Adolesc. Psychol.* 50 645–655. 10.1080/15374416.2020.1823850 33136459

[B55] McGrath R. E. Meyer G. J. (2006). When effect sizes disagree: The case of r and d. *Psychol. Methods* 11 386–401. 10.1037/1082-989X.11.4.386 17154753

[B56] McLaughlin K. A. King K. (2015). Developmental trajectories of anxiety and depression in early adolescence. *J. Abnorm. Child Psychol.* 43 311–323. 10.1007/s10802-014-9898-1 24996791 PMC4286282

[B57] Melamed N. Meizner I. Mashiach R. Wiznitzer A. Glezerman M. Yogev Y. (2013). Fetal sex and intrauterine growth patterns. *J. Ultrasound Med.* 32 35–43. 10.7863/jum.2013.32.1.35 23269708

[B58] Mendes S. L. Pinaya W. H. L. Pan P. Sato J. R. (2021). Estimating gender and age from brain structural MRI of children and adolescents: A 3D convolutional neural network multitask learning model. *Comput. Intell. Neurosci.* 2021:5550914. 10.1155/2021/5550914 34122531 PMC8172319

[B59] Miller D. J. Duka T. Stimpson C. D. Schapiro S. J. Baze W. B. McArthur M. J. (2012). Prolonged myelination in human neocortical evolution. *Proc. Natl. Acad. Sci. U. S. A.* 109 16480–16485. 10.1073/pnas.1117943109 23012402 PMC3478650

[B60] Mous S. E. Muetzel R. L. El Marroun H. Polderman T. J. van der Lugt A. Jaddoe V. W. (2014). Cortical thickness and inattention/hyperactivity symptoms in young children: A population-based study. *Psychol. Med.* 44 3203–3213. 10.1017/S0033291714000877 25065362

[B61] Naidich T. P. Ng J. C. Waselus J. D. Delman B. N. Tang C. Y. (2013). “Deep gray nuclei and related fiber tracts,” in *Imaging of the Brain*, eds Naidich T. P. Castillo N. Cha S. Smirniotopoulos J. G. (Amsterdam: Elsevier), 174–204. 10.1016/B978-1-4160-5009-4.50019-4

[B62] Nassar G. N. Leslie S. W. (2018). *Physiology, Testosterone.* Treasure Island, FL: StatPearls Publishing. 30252384

[B63] National Academies of Sciences Engineering Medicine (2022). *Measuring Sex, Gender Identity, and Sexual Orientation*, eds Becker T. Chin M. Bates N. (Washington, DC: National Academies Press). 35286054

[B64] Ngun T. C. Ghahramani N. Sánchez F. J. Bocklandt S. Vilain E. (2011). The genetics of sex differences in brain and behavior. *Front. Neuroendocrinol.* 32 227–246. 10.1016/j.yfrne.2010.10.001 20951723 PMC3030621

[B65] Nottebohm F. Arnold A. P. (1976). Sexual dimorphism in vocal control areas of the songbird brain. *Science* 194 211–213. 10.1126/science.959852 959852

[B66] Ohannessian C. M. Milan S. Vannucci A. (2017). Gender differences in anxiety trajectories from middle to late adolescence. *J. Youth Adolesc.* 46 826–839. 10.1007/s10964-016-0619-7 27889856 PMC5815170

[B67] Paus T. Keshavan M. Giedd J. N. (2008). Why do many psychiatric disorders emerge during adolescence? *Nat. Rev. Neurosci.* 9 947–957. 10.1038/nrn2513 19002191 PMC2762785

[B68] Paus T. Nawaz-Khan I. Leonard G. Perron M. Pike G. B. Pitiot A. (2010). Sexual dimorphism in the adolescent brain: Role of testosterone and androgen receptor in global and local volumes of grey and white matter. *Horm. Behav.* 57 63–75. 10.1016/j.yhbeh.2009.08.004 19703457

[B69] Paus T. Wong A. P. Syme C. Pausova Z. (2017). Sex differences in the adolescent brain and body: Findings from the saguenay youth study. *J. Neurosci. Res.* 95 362–370. 10.1002/jnr.23825 27870454

[B70] Perni S. C. Chervenak F. A. Kalish R. B. Magherini-Rothe S. Predanic M. Streltzoff J. (2004). Intraobserver and interobserver reproducibility of fetal biometry. *Ultrasound Obstet. Gynecol.* 24 654–658. 10.1002/uog.1717 15476300

[B71] Petanjek Z. Judaš M. Šimic G. Rasin M. R. Uylings H. B. Rakic P. (2011). Extraordinary neoteny of synaptic spines in the human prefrontal cortex. *Proc. Natl. Acad. Sci. U. S. A.* 108 13281–13286. 10.1073/pnas.1105108108 21788513 PMC3156171

[B72] Pletzer B. (2019). Sex hormones and gender role relate to gray matter volumes in sexually dimorphic brain areas. *Front. Neurosci.* 13:592. 10.3389/fnins.2019.00592 31275099 PMC6591487

[B73] Rey R. Josso N. Racine C. (2020). “Sexual differentiation,” in *Endotext [Internet]*, eds Feingold K. R. Anawalt B. Boyce A. Chrousos G. de Herder W. W. Dhatariya K. (South Dartmouth, MA: MDText.com, Inc).

[B74] Ritchie S. J. Cox S. R. Shen X. Lombardo M. V. Reus L. M. Alloza C. (2018). Sex differences in the adult human brain: Evidence from 5216 UK biobank participants. *Cereb. Cortex* 28 2959–2975. 10.1093/cercor/bhy109 29771288 PMC6041980

[B75] Ruigrok A. N. Salimi-Khorshidi G. Lai M. C. Baron-Cohen S. Lombardo M. V. Tait R. J. (2014). A meta-analysis of sex differences in human brain structure. *Neurosci. Biobehav. Rev.* 39 34–50. 10.1016/j.neubiorev.2013.12.004 24374381 PMC3969295

[B76] Schwärzler P. Bland J. M. Holden D. Campbell S. Ville Y. (2004). Sex-specific antenatal reference growth charts for uncomplicated singleton pregnancies at 15-40 weeks of gestation. *Ultrasound Obstet. Gynecol.* 23 23–29. 10.1002/uog.966 14970994

[B77] Sepehrband F. Lynch K. M. Cabeen R. P. Gonzalez-Zacarias C. Zhao L. D’Arcy M. (2018). Neuroanatomical morphometric characterization of sex differences in youth using statistical learning. *Neuroimage* 172 217–227. 10.1016/j.neuroimage.2018.01.065 29414494 PMC5967879

[B78] Serra-Blasco M. Radua J. Soriano-Mas C. Gómez-Benlloch A. Porta-Casteràs D. Carulla-Roig M. (2021). Structural brain correlates in major depression, anxiety disorders and post-traumatic stress disorder: A voxel-based morphometry meta-analysis. *Neurosci. Biobehav. Rev.* 129 269–281. 10.1016/j.neubiorev.2021.07.002 34256069

[B79] Shaw P. Ishii-Takahashi A. Park M. T. Devenyi G. A. Zibman C. Kasparek S. (2018). A multicohort, longitudinal study of cerebellar development in attention deficit hyperactivity disorder. *J. Child Psychol. Psychiatry* 59 1114–1123. 10.1111/jcpp.12920 29693267 PMC6158081

[B80] Sisk C. L. (2017). Development: Pubertal hormones meet the adolescent brain. *Curr. Biol.* 27 R706–R708. 10.1016/j.cub.2017.05.092 28743017

[B81] Sisk C. L. Zehr J. L. (2005). Pubertal hormones organize the adolescent brain and behavior. *Front. Neuroendocrinol.* 26 163–174. 10.1016/j.yfrne.2005.10.003 16309736

[B82] Than S. Moran C. Beare R. Vincent A. J. Collyer T. A. Wang W. (2021). Interactions between age, sex, menopause, and brain structure at midlife: A UK biobank study. *J. Clin. Endocrinol. Metab.* 106 410–420. 10.1210/clinem/dgaa847 33205159

[B83] Tomoto T. Tarumi T. Zhang R. (2023). Central arterial stiffness, brain white matter hyperintensity and total brain volume across the adult lifespan. *J. Hypertens.* 41 819–829. 10.1097/HJH.0000000000003404 36883450 PMC10079586

[B84] Urien L. Bauer E. P. (2022). Sex differences in BNST and amygdala activation by contextual, cued, and unpredictable threats. *eNeuro* 9:ENEURO.0233-21.2021. 10.1523/ENEURO.0233-21.2021 34911788 PMC8741146

[B85] Vijayakumar N. Youssef G. J. Allen N. B. Anderson V. Efron D. Hazell P. (2021). A longitudinal analysis of puberty-related cortical development. *Neuroimage* 228:117684. 10.1016/j.neuroimage.2020.117684 33385548

[B86] Whitwell J. L. Crum W. R. Watt H. C. Fox N. C. (2001). Normalization of cerebral volumes by use of intracranial volume: Implications for longitudinal quantitative MR imaging. *AJNR Am. J. Neuroradiol.* 22 1483–1489. 11559495 PMC7974589

[B87] Witte A. V. Savli M. Holik A. Kasper S. Lanzenberger R. (2010). Regional sex differences in grey matter volume are associated with sex hormones in the young adult human brain. *Neuroimage* 49 1205–1212. 10.1016/j.neuroimage.2009.09.046 19796695

[B88] World Health Organization [WHO] (2007). *WHO Child Growth Standards: Head Circumference-for-Age, Arm Circumference-for-Age, Triceps Skinfold-for-Age and Subscapular Skinfold-for-Age: Methods and Development.* Geneva: World Health Organization.

[B89] Wright E. C. Luo P. X. Zakharenkov H. C. Serna Godoy A. Lake A. A. Prince Z. D. (2023). Sexual differentiation of neural mechanisms of stress sensitivity during puberty. *Proc. Natl. Acad. Sci. U. S. A.* 120:e2306475120. 10.1073/pnas.2306475120 37847733 PMC10614610

[B90] Wyburd M. K. Dinsdale N. Kyriakopoulou V. Venturini L. Uus A. Matthew J. (2024). EP04. 02: Fetal brain imaging in 3D: A direct comparison between same day MRI and ultrasound volumetric measures. *Ultrasound Obstet. Gynecol.* 64:129. 10.1002/uog.2809138808587

[B91] Wycoco V. Shroff M. Sudhakar S. Lee W. (2013). White matter anatomy: What the radiologist needs to know. *Neuroimaging Clin. N. Am.* 23 197–216. 10.1016/j.nic.2012.12.002 23608685

[B92] Yeo G. S. Qi M. Du R. Mahavadi P. L. Yung C. F. Thoon K. C. (2017). Gender-specific reference charts of fetal head circumference in a singaporean population. *Ann. Acad. Med. Singap.* 46 367–373. 29177365

[B93] Yu Y. Cui H. Q. Haas S. S. New F. Sanford N. Yu K. (2024). Brain-age prediction: Systematic evaluation of site effects, and sample age range and size. *Hum. Brain Mapp.* 45:e26768. 10.1002/hbm.26768 38949537 PMC11215839

[B94] Zong X. N. Li H. (2013). Construction of a new growth references for China based on urban Chinese children: Comparison with the WHO growth standards. *PLoS One* 8:e59569. 10.1371/journal.pone.0059569 23527219 PMC3602372

