# Supplementary material for: Sex differences in global metrics of brain size across the lifespan
Source: Front Neurosci. 2026 Feb 27;20:1646144. doi: 10.3389/fnins.2026.1646144 (PMC12982445; doi:10.3389/fnins.2026.1646144)
Supplement: Supplementary file 1 [file Data_Sheet_1.docx]

Supplementary Material

# Supplementary Figures


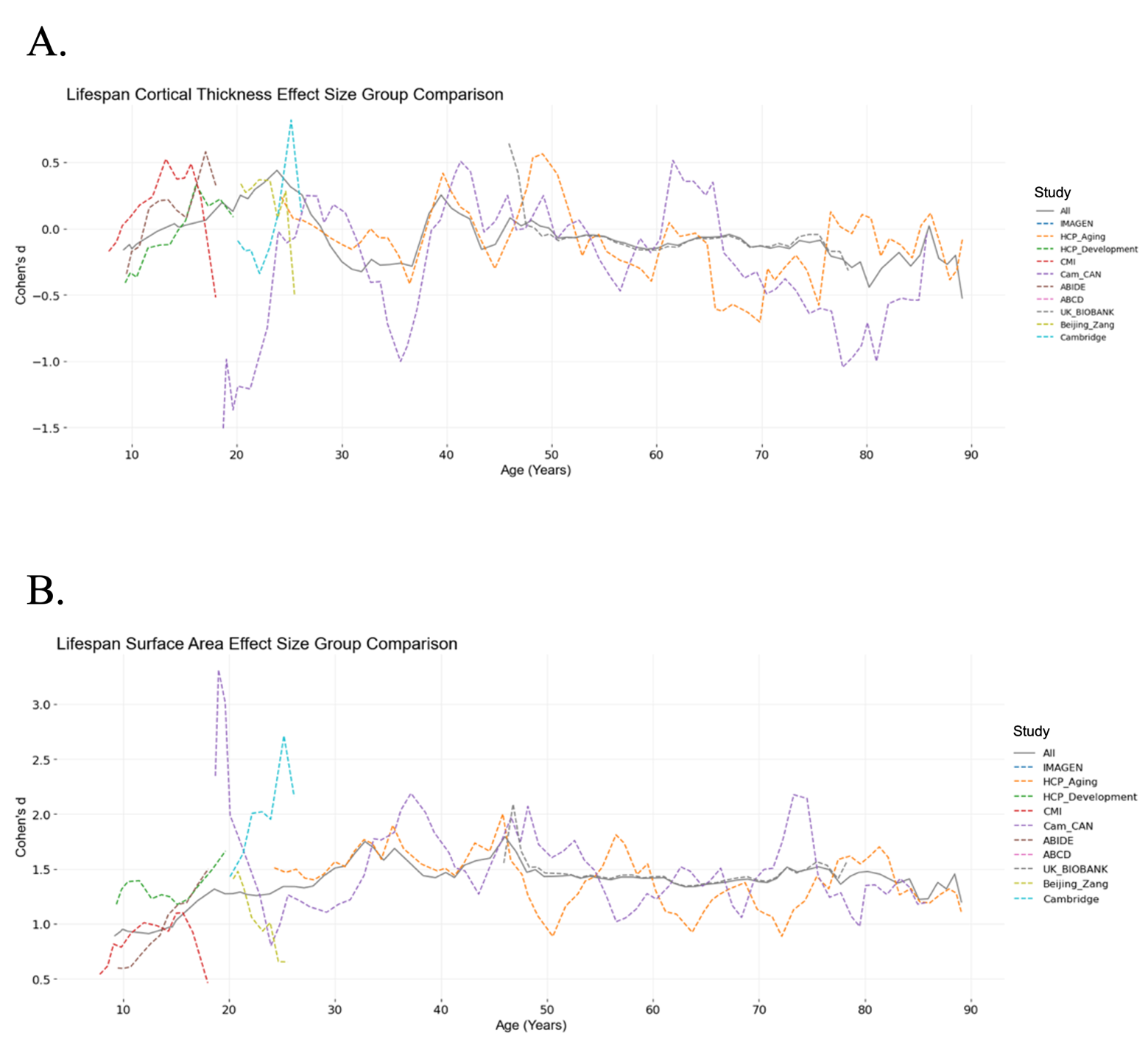


**Supplementary Figure 1. Lifespan Morphometry by Study.** (A) Lifespan cortical thickness effect size of individual studies is plotted against age range. (B) Lifespan surface area effect size of individual studies is plotted against age range. The solid line represents all MRI data aggregated, while the datasets listed contain the major contributing datasets to the overall trend. It is challenging to distinguish which individual data sources affect most directly CVES. However, when breaking down the two factors of CVES (cortical thickness effect size and surface area effect size into effect size computations from individual contributing studies, the discernable result is not surprising. The contributing study which made up over 50% of all MRI data, the UK Biobank, demonstrates trends in cortical thickness and surface area effect size which most closely resemble trends for these effect size computations across all data sources (Figure 3C-D).


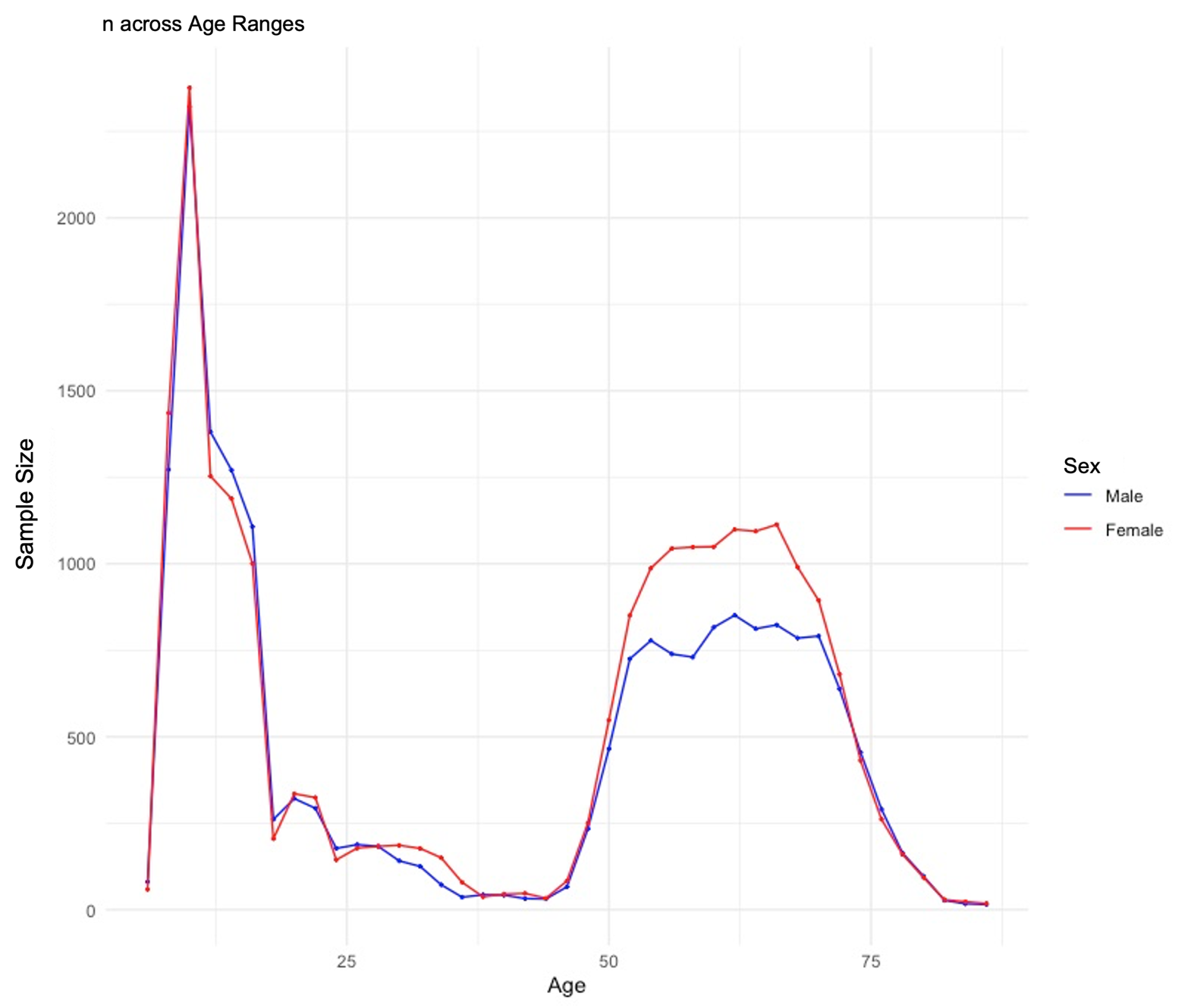


**Supplementary Figure 2. MRI n across Age Ranges.** Supplementary Figure 2. MRI Sample Size across Age Ranges. Sample size for males and females is plotted against “window” or a subset of data in the sliding window analysis. A particular subset of data is demarcated on the x-axis by its “window median” (e.g. “2” in the window from 0-4 years). There is an exponential increase in sample size at the age 10 window marker, and an exponential decrease in sample size at the age 18 window marker. Relatively small sample sizes of <250 are seen in very early life, between 25 and 45 years, and after 75.
